# Cystic echinococcosis in Nigeria: first insight into the genotypes of *Echinococcus granulosus* in animals

**DOI:** 10.1186/s13071-019-3644-z

**Published:** 2019-08-07

**Authors:** John Asekhaen Ohiolei, Hong-Bin Yan, Li Li, Abdullahi Alhaji Magaji, Joshua Luka, Guo-Qiang Zhu, Clement Isaac, Manfred Ebube Odoya, Yan-Tao Wu, Mughees Aizaz Alvi, Rosline James Muku, Bao-Quan Fu, Wan-Zhong Jia

**Affiliations:** 1 State Key Laboratory of Veterinary Etiological Biology/National Professional Laboratory of Animal Hydatidosis, Lanzhou Veterinary Research Institute, CAAS, Lanzhou, 730046 Gansu P. R. China; 2 Department of Zoology, Faculty of Life Sciences, Ambrose Alli University, Ekpoma, Nigeria; 3 Department of Veterinary Public Health and Preventive Medicine, Faculty of Veterinary Medicine, Usmanu Danfodiyo University, Sokoto, Nigeria; 4 Department of Veterinary Parasitology and Entomology, Faculty of Veterinary Medicine, University of Maiduguri, Maiduguri, Nigeria

**Keywords:** Cystic echinococcosis, Haplotypes, Genetic variation, *Echinococcus canadensis*, Phylogeny

## Abstract

**Background:**

Cystic echinococcosis (CE) is a zoonosis caused by cestodes of *Echinococcus granulosus* (*sensu lato*) complex. In Nigeria, reports on the prevalence of CE, although limited, have been found to vary with location and host with higher prevalence and fertility rate observed in camels than other livestock. Until now, information regarding the molecular characteristics, genetic population structure, and genotypes of *Echinococcus* is lacking. Therefore, this study was aimed at addressing these gaps in knowledge.

**Methods:**

We describe the genetic status of 31 *Echinococcus* isolates collected from slaughtered livestock (camels, cattle and goats) based on the full-length mitochondrial cytochrome c oxidase subunit 1 (*cox*1) and NADH dehydrogenase subunit 1 (*nad*1) genes.

**Results:**

The resulting nucleotide sequences *via* the NCBI BLAST algorithm and Bayesian phylogeny of *cox*1 and *cox*1*–nad*1 genes using MrBayes v.3.1.2 showed that all isolates were clearly *E. canadensis* (G6/G7) and were 99–100% identical to previously reported G6/G7 haplotypes across Europe, Asia, North and East Africa.

**Conclusions:**

Although, the G1 genotype is believed to be responsible for the majority of global CE burden, reports from a number of West African countries including Nigeria suggest that *E. canadensis* G6/G7 genotype could be the major causative agent of CE in the subregion. This study provides for the first time insight into the genetic population structure of *Echinococcus* species as well as implications for CE control in Nigeria.

**Electronic supplementary material:**

The online version of this article (10.1186/s13071-019-3644-z) contains supplementary material, which is available to authorized users.

## Background

Cystic echinococcosis (CE) is caused by larval stage metacestodes of *Echinococcus granulosus* (*sensu lato*) of which canids are definitive hosts while a wide range of domestic ungulates act mainly as intermediate hosts. Globally, the economic losses due to the burden of CE have been estimated as reaching billions of US dollars annually [1, 2]. CE is common in Africa, especially but not limited to northern and eastern African countries [2–7]. Additionally, high genetic diversity of *Echinococcus* species has been observed [7]. In most West African countries, data on the prevalence and genetic diversity of *Echinococcus* species is broadly lacking as information of the genotypes is only available for a few countries [8–11]. Species of *E. granulosus* (*s.l.*) have shown considerable variation within their mitochondrial DNA resulting in categorization into *E. granulosus* (*sensu stricto*) (G1, G3), *E. equinus* (G4), *E. ortleppi* (G5), *E. canadensis* (G6-G10), as well as *E. felidis* [12]. However, there are pending controversies regarding the taxonomy of *E. canadensis* group as some authors have suggested that genotypes G6/G7 be categorised as *E. intermedius*, while genotypes G8 and G10 as *E. canadensis* [12–14].

Therefore, knowledge of the identity of species and their genotypes from a range of hosts in a given location could prove useful in understanding and appreciating the disease dynamics; this could guide towards designing effective control and prevention schemes [15, 16].

In Nigeria, reports during the last three decades have shown a high prevalence of CE in livestock, particularly in the northern region [17, 18], with the highest prevalence and fertility rate in camels [18]. The seeming impact of CE infection on livestock production is yet to be evaluated in Nigeria. Therefore, in furthering this course, information regarding species variation and genotypes responsible for infection could be crucial. To our knowledge, we thus provide for the first time an insight into the genetic population structure and phylogenetic relationship of *Echinococcus* species in Nigeria.

## Methods

### Study area

**Fig. 1:**
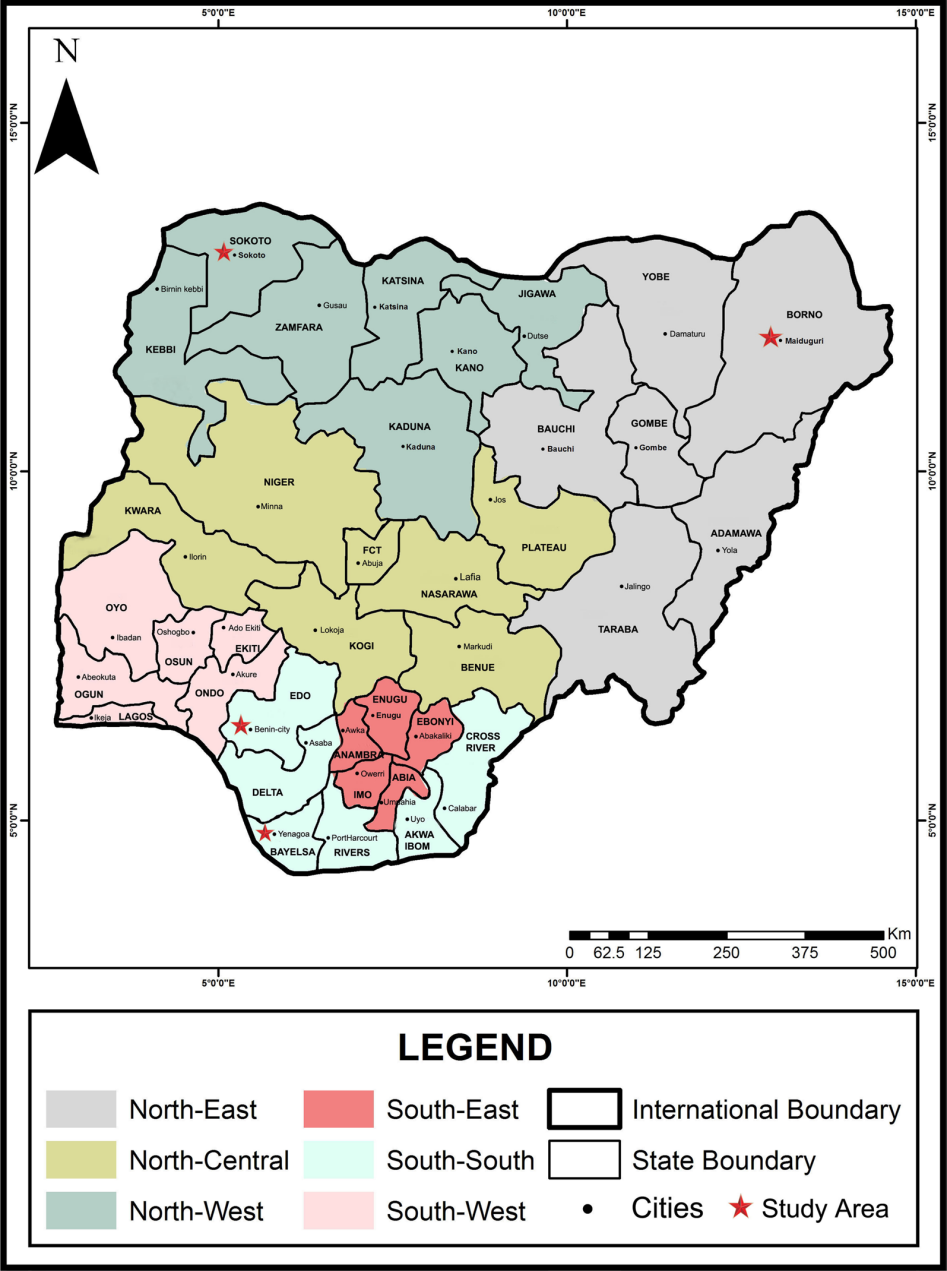
Map of Nigeria showing the six geopolitical zones, states and study areas

Nigeria is in West Africa, has a population of over 180 million, and comprises 36 states and a Federal Capital Territory (Abuja). These states are grouped into six geopolitical zones (North-East, North-Central, North-West, South-East, South-South and South-West) based on ethnicity and common history/ancestry. The vegetation cover is mostly rainforest in the south and savannah in the north. Owing to its favorable climate, it supports large biodiversity and is thus endemic for a number of parasitic zoonoses including cystic echinococcosis. Sokoto and Maiduguri are capital cities located in the North-West and North-East zones of Nigeria, respectively, while Benin-city and Yenagoa are situated in the South-South zone (Fig. 1).

### Parasite material

A total of 1592 heads of livestock [camels (*n *= 118), cattle (*n *= 856), sheep (*n *= 300) and goats (*n *= 318); Table 1] were investigated *post-mortem* for infection with *Echinococcus* spp. within a two-month period (November and December 2018) in the following locations: Sokoto modern abattoir, Sokoto state; Maiduguri abattoir, Borno state; Aduwawa cattle market in Benin-city, Edo state; and Yenagoa abattoir in Bayelsa state. Furthermore, faecal samples from privately-owned dogs (*n *= 51) within the city metropolis, and lions (*n *= 3) and a hyena (*n *= 1) from a zoological garden in Benin-city (Table 1) were examined for *Echinococcus* eggs using a modified sucrose flotation method and polymerase chain reaction after DNA extraction from faeces (QIAamp Fast DNA Stool Mini-kit; Qiagen, Hilden, Germany).

**Table 1:** Number of animals examined, prevalence, and characteristics of isolates found in study areas

	Sokoto					Maiduguri		Yenagoa		Benin-city				
	Camels (*n *= 118)	Cattle (*n *= 454)	Goats (*n *= 120)	Sheep (*n *= 120)	Dogs (*n *= 23)	Sheep (*n *= 180)	Goats (*n *= 180)	Dogs (*n *= 12)	Cattle (*n *= 360)	Dogs (*n *= 16)	Cattle (*n *= 42)	Goats (*n *= 18)	Lions (*n *= 3)	Hyena (*n *= 1)
No. infected (prevalence, %)	23 (19.49)	4 (0.88)	–	–	–	–	1 (0.56)	–	–	–	–	–	–	–
Age range (years)	8–10	2–5	1–2	1–2	0.5–5	0–2	0–2	0.5–4	2–5	0.5–4	2–5	1.2–2	2–11	> 5
Isolates per organ/site														
Lungs	25	1	–	–	ne	–	–	ne	–	ne	–	–	ne	ne
Liver	1	3	–	–	ne	–	1	ne	–	ne	–	–	ne	ne
Spleen	1	–	–	–	ne	–	–	ne	–	ne	–	–	ne	ne
Heart	–	–	–	–	ne	–	–	ne	–	ne	–	–	ne	ne
Faeces	ne	ne	ne	ne	–	ne	ne	–	ne	–	ne	ne	–	–
% of animals with fertile cyst	100	50	–	–	–	–	0	–	–	–	–	–	–	–

*Abbreviation*: ne, not examined

### DNA extraction, amplification, and sequencing of isolates

Prior to DNA extraction, collected cysts were first cleaned with 75% ethanol and germinal layers were removed and repeatedly washed with phosphate-buffered solution (PBS). Protoscoleces from fertile cysts were also washed in PBS and stored until use. DNA was extracted from germinal layers and protoscoleces. Briefly, a portion of the germinal layer from each isolate was crushed in liquid nitrogen followed by total genomic DNA extraction using a Qiagen Blood and Tissue Kit (Qiagen) according to the manufacturer’s instructions. Afterwards, PCR was conducted in a 25 µl final volume using 2 mM MgCl_2_, 0.2 mM dNTPs, 5 µl of 5× *Taq* buffer, 10 pmol of each primer, 0.5 µl of Ex *Taq* DNA polymerase (5 U/µl; TaKaRa, Kusatsu, Japan), 0.5 μl of genomic DNA extract (~20–200 ng) and RNAse free water to make up the final volume. The PCR conditions were as follows: initial denaturation at 95 °C for 5 min; 35 cycles of denaturation at 95 °C for 30 s, annealing at 55 °C for 40 s and elongation at 72 °C for 60–90s; and a final extension step at 72 °C for 10 min.

Amplification of the complete mitochondrial *nad*1 gene (894 bp) using forward primer (5′-ATT ATA GAA AAT TTT CGT TTT ACA CGC-3′) and reverse primer (5′-ATT CAC AAT TTA CTA TAT CAA AGT AAC C-3′), and complete *cox*1 gene (1608 bp) using forward primer (5′-ATT ATA GAA AAT TTT CGT TTT ACA CGC-3′) and reverse primer (5′-AAG CAT GAT GCA AAA GGC AAA TAA ACC-3′) [19] was carried out for all isolates. In addition to the above primers, previously designed primers [20] were also used to detect the presence of *Echnococcus* in faecal samples. PCR products were detected in a 1.5% (w/v) agarose gel stained with GelRed^TM^. Five microliters of the amplicon was used for visualization while the rest was sequenced in an ABI3730xl DNA Analyser (Beijing Tsingke Biotechnology Co., Ltd., Beijing, China).

### Molecular analysis

DNA sequences were viewed and manually corrected for any misread nucleotides using BioEdit software [21]. Resulting gene sequences were aligned with BioEdit [21] and the identity of each isolate was confirmed with their nucleotide sequence in the GenBank database using the NCBI BLAST algorithm (https://blast.ncbi.nlm.nih.gov/Blast.cgi). Nucleotide and haplotype diversity indices were estimated in DnaSP v.6 [22]. Median-joining network [23] was inferred based on the sequences of mitochondrial *cox*1, *nad*1, and *cox*1*–nad*1 genes using PopART (http://popart.otago.ac.nz). Population neutrality indices; Tajima’s D [24] and Fu’s Fs [25] were calculated using DnaSP v.6 [22]. Bayesian phylogeny was inferred based on the *cox*1 and *cox*1–*nad*1 dataset using MrBayes v.3.1.2. Markov Chain Monte Carlo (MCMC) sampling was used to assess the posterior distribution of parameters with a chain length of 2,000,000 states, and 25% was discarded as burn-in. Parameters were logged every 1000 states. TreeView v.1.6.6. (http://taxonomy.zoology.gla.ac.uk/rod/treeview.html) was used to display tree.

## Results

Overall, of livestock examined, 19.49% (23/118) of camels, 0.47% (4/856) of cattle, 0.31% (1/318) of goats and 0% (0/300) of sheep were positive of 32 hydatid cysts from lungs, liver and spleen (Table 1). No infection was detected in all faecal samples examined. Out of the 32 isolates, 31 were successfully amplified for *cox*1 and *nad*1 mitochondrial genes and were identified as *E. canadensis* G6/G7 genotype using the NCBI BLAST algorithm with 99–100% identity. Analysis of the resulting sequences showed a total of 4 mutation sites (*cox*1= 3, *nad*1= 1) (Table 2) with 3 parsimony informative sites (*nad*1= 1, *cox*1= 2) (Table 3). No insertions or deletions were observed.

**Table 2:** Variation sites of *cox*1 and *nad*1 genes of *Echinococcus canadensis* (G6/G7) haplotypes found in Sokoto and Maiduguri, Nigeria

Haplotype	Origin	No. of isolates	*cox*1 mutation site			Haplotype	Origin	No. of isolates	*nad*1 mutation site
			79	510	1171				354
H1	Sokoto, Maiduguri	24	G	C	A	H1	Sokoto, Maiduguri	28	C
H2	Sokoto	3	–	T	–	H2	Sokoto	3	T
H3	Sokoto	1	T	–	–				
H4	Maiduguri	3	–	–	G

**Table 3:** Diversity and neutrality indices for *Echinococcus canadensis* (G6/G7) populations from Sokoto and Maiduguri, Nigeria

Indices	*cox*1 (1608 bp)	*nad*1 (894 bp)	*cox*1–*nad*1 (2502 bp)
No. of isolates	31	31	31
No. of mutations	3	1	4
Parsimony informative sites	2	1	3
No. of haplotypes	4	2	5
Haplotype diversity (Hd)	0.3935	0.181	0.529
Nucleotide diversity (π)	0.00026	0.00740	0.00024
Tajima’s D (*P-*value)	− 1.00957	− 0.42924	− 1.00361
Fu’s Fs	− 1.551	0.009	− 1.926

### Haplotype network of *Echinococcus canadensis*

**Fig. 2:**
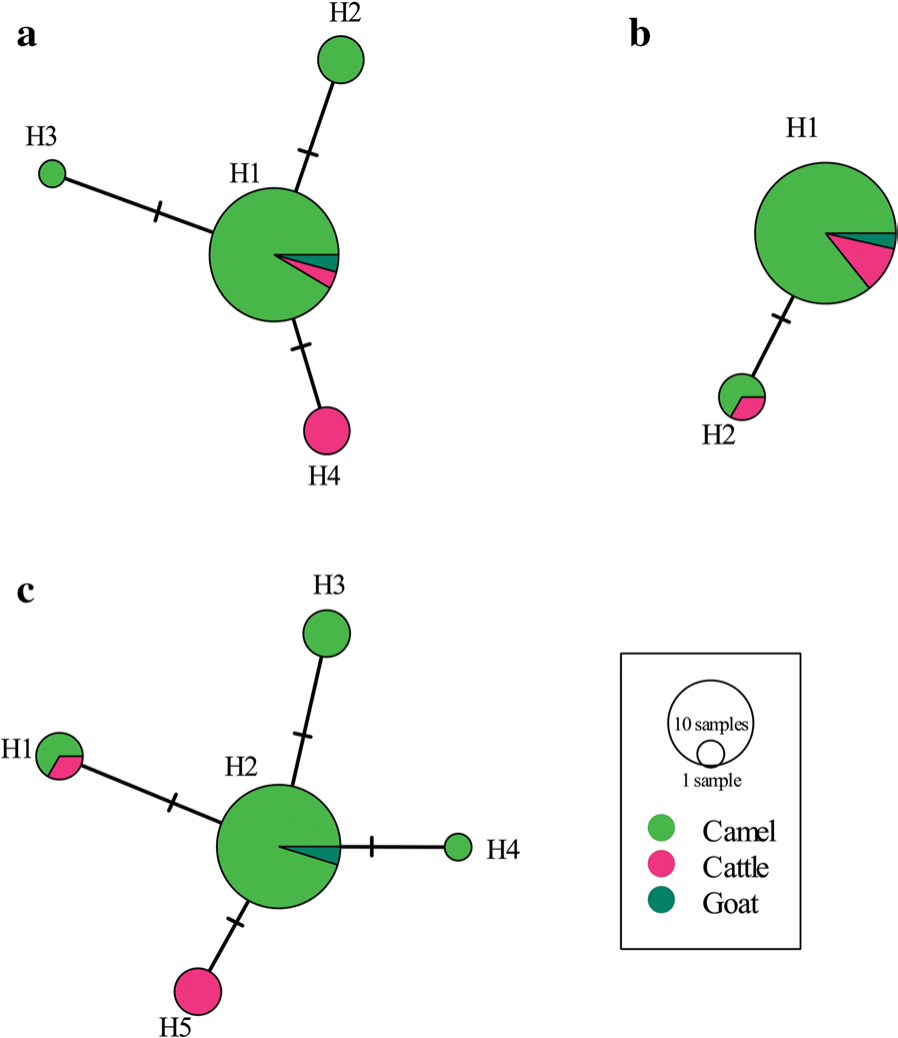
Median-joining network of *Echinococcus canadensis* G6/G7 haplotypes based on the *cox*1 (1608 bp) (**a**), *nad*1 (894 bp) (**b**), and *cox*1–*nad*1 (2502 bp) (**c**) mitochondrial genes

Among the 31 isolates, 4 and 2 haplotypes were found for *cox*1 and *nad*1 genes, respectively (Fig. 2a, b). The hosts and geographical origins of the isolates are shown in Table 1 (see Additional file 1: Figure S1 for the haplotype geographical location). Analysis of the concatenated *cox*1–*nad*1 (2502 bp) sequences showed 5 distinct haplotypes (Fig. 2c) with haplotype H2 found in both zones (North-West and North-East), constituting about 67.74% of the entire *E. canadensis* population. Additionally, 3/4 of the cattle isolates (Sokoto, North-West zone) formed a distinct haplotype (H5) (Fig. 2c). Representative *cox*1 and *nad*1 haplotype sequences from this study have been deposited in the GenBank database under the accession numbers MN025261–MN025264 (*cox*1), and MN025265 and MN025266 (*nad*1).

### Neutrality and diversity indices

The diversity and neutrality indices for the entire *E. canadensis* population were calculated based on the sequences of *cox*1, *nad*1, and *cox*1–*nad*1 genes and are presented in Table 3. Overall, *cox*1–*nad*1 haplotype diversity (Hd) and nucleotide diversity (π) were 0.529 and 0.00024, respectively, while Tajima’s *D* and Fu’s Fs were negative and insignificant for the entire population (Table 3).

### Phylogenetic analysis

**Fig. 3:**
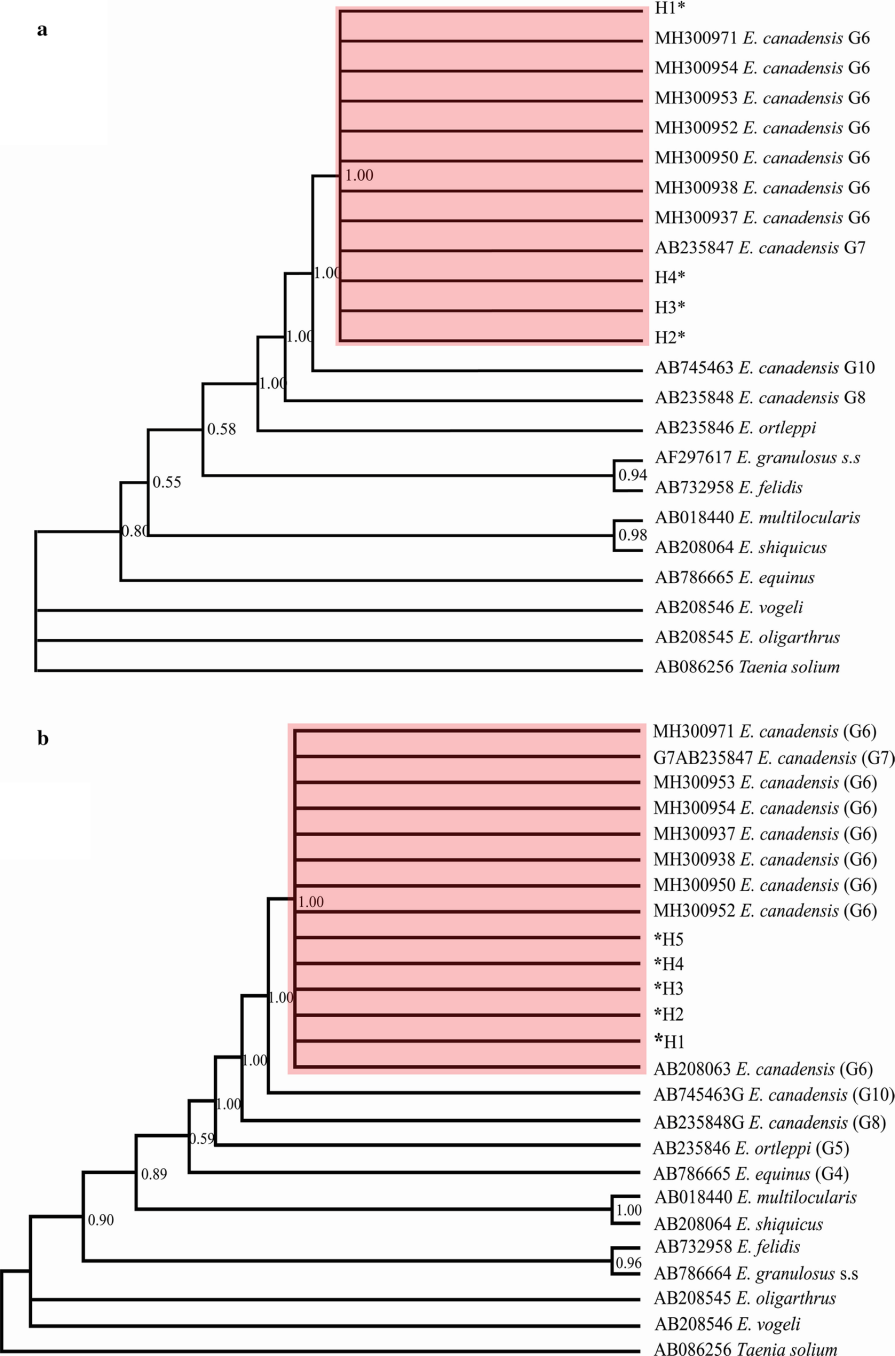
Bayesian phylogeny of Nigerian *Echinococcus canadensis* G6/G7 inferred from the *cox*1 (1608 bp) gene (**a**) and *cox*1–*nad*1 (2502 bp) (**b**) concatenation. Red= *Echinococcus canadensis* G6/G7 cluster. Posterior probability values are depicted at the nodes. *Indicates haplotypes representing isolates from this study: *cox*1 haplotypes: MN025261–MN025264 (H1–H4); *cox*1–*nad*1 haplotypes: MN025261, MN025266 (H1) MN025261, MN025265 (H2) MN025262, MN025265 (H3) MN025263, MN025265 (H4) MN025264, MN025265 (H5)

The Bayesian phylogeny based on sequences of *cox*1 and *cox*1–*nad*1 mitochondrial genes placed all Nigerian *E. canadensis* G6/G7 isolates in the same clade with other G6/G7 genotype from different hosts and countries retrieved from GenBank (Fig. 3a, b).

## Discussion

In Africa, *E*. *canadensis* (G6/G7) is the second leading cause of CE after *E. granulosus* (*s.s.*) and seems to be the major cause of CE in some countries [26, 27]. Its prevalence is also high in countries like Sudan and Egypt [26–30], with documented evidence in Mali and Mauritania [9, 10] where the population of the suitable intermediate host is high [31]. Similarly, the G6/G7 genotype has also been reportedly common in Middle East countries and Europe [32–34]. The peculiarity of this genotype to these countries could possibly have resulted from the ancient trade relationship with countries in northern Africa when camelid hosts served as major means of overland transportation. Although pigs also serve as suitable intermediate host for the G6/G7 genotype, the dearth of data on this intermediate host across Africa limits a clear description of the role of pigs in the epidemiology of the G6/G7 genotype.

The CE prevalence reported in this study showed some degree of variation among intermediate hosts as camels had a higher rate of infection. Previous studies have reported similar results implicating camels as most susceptible. In some areas, prevalence has been found to reach as high as 70% in camels and 40% in other livestock [18, 35, 36], although lower prevalence has also been documented in other locations [37, 38]. The high fertility rate observed in camels suggests that they play a significant epidemiological role in the transmission of CE in Nigeria, especially in the North-West and North-East zones. On the other hand, the situation in the South-West and South-South cannot be completely described at the moment due to the dearth of data. Previously, studies on CE in the Niger Delta areas (South-South) conducted in the last two decades reported prevalences ranging between 24.4–55.9% among livestock and over 80% in dogs [39]. Since then, follow-up studies have not been carried out to update CE epidemiological data in this region. A similar situation is the case for the South-West region where previous studies within the said period estimated CE prevalence at 28% in sheep and goats [40]. Currently, reports from the aforementioned region are seroprevalence estimations [41] which makes the CE status in both region unclear and difficult to describe because of the inability of current serological tools to discriminate between strains/genotypes and/or cross-reactivity with other *Taenia* species [42–44]. The absence of CE in the examined livestock from two South-South cities as reported in this study may not completely reflect the CE situation in the region. However, a possible explanation for this observation could relate to (i) the absence or extremely low population of camels of which they are potentially known to maintain the infection; (ii) the fact that majority of slaughtered animals for consumption in the southern part of Nigeria are reared in the north as only seemingly healthy livestock are transported to the south (since vendors need to be sure that such livestock can withstand the stress of road transportation); and (iii) the different climatic conditions and vegetation cover between northern and southern Nigeria which could impact egg survival. Nonetheless, a detailed longitudinal study in the south compared to the north will be vital in appraising the climatic and transboundary effect on CE prevalence across zones.

In this study, we confirm the presence of the ‘camel’ strain of *E. granulosus* in camelid and non-camelid hosts (cattle and goats) from the northern part of Nigeria. This report is also in line with previous observations regarding the preponderance of *E. canadensis* G6/G7 in the West African region. For example, the ‘camel’ strain has been implicated in camels, cattle and humans from Mauritania [8, 9, 11], with a similar finding in a dog from Mali [10].

The low nucleotide and haplotype diversity observed in this study is comparable to previous reports of *E. canadensis* G6/G7 population in some countries [33, 34, 45]. Additionally, a lower number of haplotypes was found among the 31 isolates using *cox*1, *nad*1 and *cox*1–*nad*1 genes when compared to haplotypes recorded in Mongolia [46], but similar to the number of haplotypes reported from Hungary [45]. Our result is also in conformity with previous observations of low polymorphism frequently exhibited by *E*. *canadensis* G6/G7 populations in most parts of the world [33, 34]. The haplotype and nucleotide diversity based on the 2502 bp *cox*1–*nad*1 genes when compared to global G6/G7 clusters recently reported by Addy et al. [34], were more similar to the French G6/7 population than those of northern and eastern African countries and other geographical locations.

The network analysis based on *cox*1–*nad*1 concatenated sequences showed that the major haplotype (H2) constituted 67.74% of the entire *E. canadensis* population and had a 100% homology with other widespread haplotypes in camels from Mauritania, Sudan and Iran [47], goat isolates from Sudan and Argentina [47], and human isolates from Kenya [47]. It was also identical to the *Gmon* haplotype of human origin from Mongolia [47] and the *cox*1 human isolates from Russia [48]. The same haplotype, when compared to the recent global G6/G7 clusters, showed 99.78–99.94% similarity to haplotypes from Kenyan camels, goats, dogs and humans; Sudanese camels and humans; French pigs; and Iranian camels and humans [34]. However, the *cox*1 of H3 and *nad*1 of H4 were found to be 100% identical to the haplotypes of camel origin from Kenya: Ec04 (GenBank: KX010833) and Ec01 (GenBank: KX010873), respectively.

The similarity observed between Nigerian isolates and those from elsewhere agrees with the initial proposition of non-geographical distinction among G6/G7 haplotypes [34]. This observation was further corroborated by the clustering of the Nigerian isolates with isolates from other hosts and locations as evident in the Bayesian phylogeny of the *cox*1 and *cox*1–*nad*1 concatenated sequences.

Furthermore, to understand the role played by wildlife in the transmission and maintenance of CE, we examined captive lions and hyena for the possibility of infection with *Echinococcus* since they fed on carcasses of goats/sheep. Clearly, no positive case was seen. However, it will be important in the future to carry out an extensive investigation on both captive and untamed wildlife so as to establish/understand the possible nature of the interface between domestic and wild animals in the transmission of CE across the country.

## Conclusions

Our findings confirm camels as the major intermediate host responsible for the maintenance of CE in Nigeria. Although the G1 genotype is responsible for the majority of global CE burden, the case in the West African region following existing reports and our study suggest that the *E. canadensis* G6/G7 genotype due to the large involvement of camels may be the dominant species responsible for CE in the region. To our knowledge, we provide for the first time insights on the genotype/species of *Echinococcus* infecting livestock and identify *E. canadensis* G6/G7 genotype as the major cause of CE infection as well as establish their phylogenetic relationship with other isolates from different hosts/locations. This information could serve as a baseline for further studies. Regardless of the limited sample size analysed in this study, it has enriched CE data for Nigeria and indeed West Africa. We thus suggest that in the future, molecular studies should cover states in Nigeria that are yet to be investigated in order to provide robust data for control.

## Additional file

**Additional file 1: Figure S1.** *cox*1–*nad*1 haplotypes geographical distribution. H2 was found in both zones whereas other haplotypes were present only in Sokoto, northwestern Nigeria.

## Data Availability

All data supporting the conclusions of this article are included in the article and its additional file. Representative nucleotide sequences of *cox*1 and *nad*1 genes from the present study are available in the GenBank database under the Accession Numbers MN025261–MN025266.

## Abbreviations

WHO: World Health Organization
bp: base pair or base pairs
PCR: polymerase chain reaction
DNA: deoxyribonucleic acid
*cox*1: cytochrome c oxidase subunit 1
*nad*1: NADH dehydrogenase subunit 1
CE: cystic echinococcosis
MCMC: Markov Chain Monte Carlo
Hd: haplotype diversity
