# Supplementary figures and images for: Cystic echinococcosis in Nigeria: first insight into the genotypes of Echinococcus granulosus in animals
Source: Parasit Vectors. 2019 Aug 7;12:392. doi: 10.1186/s13071-019-3644-z (PMC6686243; doi:10.1186/s13071-019-3644-z)

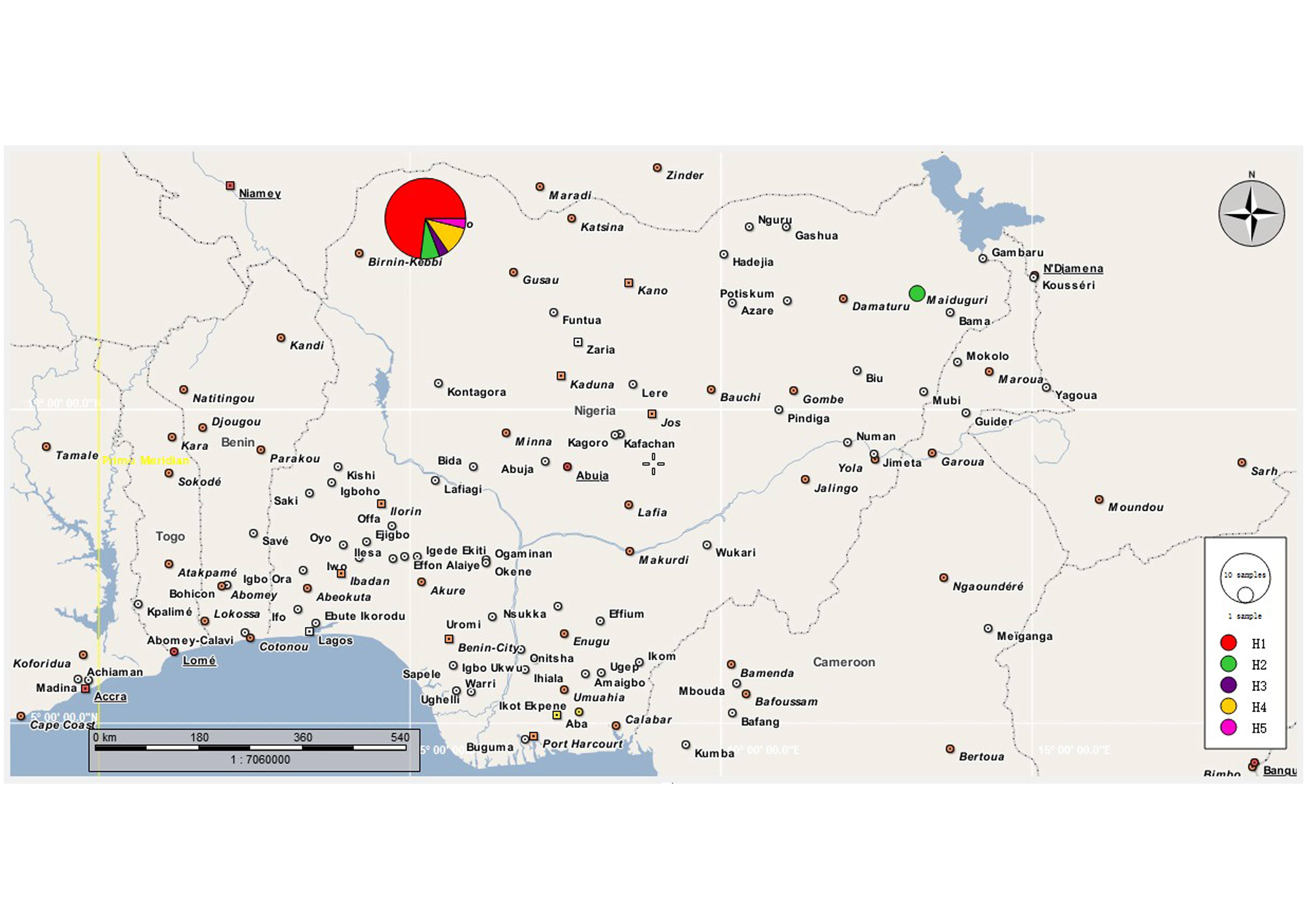

Supplement: Supplementary file 1 — Additional file 1: Figure S1. cox1–nad1 haplotypes geographical distribution. H2 was found in both zones whereas other haplotypes were present only in Sokoto, northwestern Nigeria. [file 13071_2019_3644_MOESM1_ESM.jpg]
